# Arsenic Monitoring in Water by Colorimetry Using an Optimized Leucomalachite Green Method

**DOI:** 10.3390/molecules24020339

**Published:** 2019-01-18

**Authors:** Annija Lace, David Ryan, Mark Bowkett, John Cleary

**Affiliations:** 1 EnviroCORE, Department of Science and Health, Institute of Technology Carlow, Kilkenny Road, R93 V960 Co. Carlow, Ireland; David.Ryan@itcarlow.ie (D.R.); John.Cleary@itcarlow.ie (J.C.); 2 TE Laboratories Ltd. (TelLab), Loughmartin Business Park, Tullow, R93 N529 Co. Carlow, Ireland; mbowkett@tellab.ie

**Keywords:** arsenic, colorimetric methods, environmental monitoring, leucomalachite green, microfluidics

## Abstract

Arsenic contamination of drinking water is a global concern. Standard laboratory methods that are commonly used for arsenic detection in water, such as atomic absorption spectroscopy and mass spectroscopy, are not suitable for mass monitoring purposes. Autonomous microfluidic detection systems combined with a suitable colorimetric reagent could provide an alternative to standard methods. Moreover, microfluidic detection systems would enable rapid and cost efficient in situ monitoring of water sources without the requirement of laborious sampling. The aim of this study is to optimize a colorimetric method based on leucomalachite green dye for integration into a microfluidic detection system. The colorimetric method is based on the reaction of arsenic (III) with potassium iodate in acid medium to liberate iodine, which oxidizes leucomalachite green to malachite green. A rapid colour development was observed after the addition of the dye. Beer’s law was obeyed in the range between 0.07–3 µg mL^−1^. The detection limit and quantitation limit were found to be 0.19 and 0.64 µg mL^−1^, respectively.

## 1. Introduction

Arsenic contamination of groundwater and surface water is a major issue in certain regions of the world [1]. Arsenic and its compounds are toxic and can cause serious health effects [2]. Human exposure to arsenic arises through consumption of arsenic contaminated food and water. Chronic exposure to high concentrations of arsenic can cause severe health implications, collectively known as arsenicosis [3]. Some of the symptoms of arsenicosis include skin lesions, nervous system disorders, gastrointestinal problems and various types of cancers [4,5].

Inorganic arsenic is naturally present at high levels in the groundwater of several countries, including India [6,7] Pakistan [8], China [9], Vietnam [10] and several parts of the United States [11,12,13]. Arsenic contamination of ground water in Bangladesh is one of the most serious examples of chronic arsenic exposure [14]. Arsenic concentrations in drinking water in Bangladesh far exceed the World Health Organization’s (WHO) maximum permissible limit of 10 μg L^−1^ [15]. In some tube wells arsenic concentrations as high as 2500 μg L^−1^ have been detected [16].

In order to improve and monitor the environmental quality of water, reliable and good quality information is needed. Analytical methods capable of detecting arsenic at low concentrations are usually based on sophisticated laboratory instrumentation [17]. Atomic absorption spectroscopy (AAS), induced coupled plasma atomic emission spectroscopy (ICP-AES), X-ray fluorescence, and atomic fluorescence spectroscopy are examples of sensitive and selective methods used for arsenic detection and analysis [18]. While high quality data is obtained from these methods, the cost of analyzing water samples is significant due to the manpower and instrumentation requirements and the overall cost of analysis. Therefore, these powerful laboratory methods are not suitable for routine, high frequency arsenic monitoring [19].

Given the challenges associated with rapid and affordable arsenic detection, much research has been carried out for alternative method development [20,21,22,23,24]. Because of their small size microfluidic detection systems have many advantages over traditional laboratory-based analytical techniques [25]. The small size enables development of compact and portable sensing systems, sometimes referred to as point of need or point of care systems, that can be directly applied at a sampling site without the need for sample collection, transport, or storage [26]. The micro scale enables fast reaction time which in turn results in high sample throughput. As small sample size is required when working on the microscale, reagent consumption and waste generation are minimized. These properties of microfluidic detection systems make them suitable for development of autonomous in situ water monitoring sensors [27].

Microfluidic detection systems have been developed for phosphate [28], nitrate [29], ammonia [30] and pH [31] monitoring in water. Nevertheless, very few commercially available microfluidic detection systems have been developed for heavy metal monitoring in water. The low limit of detection for arsenic and other heavy metals poses a key challenge for development of microfluidic sensing systems for drinking water applications. Interfering substances, turbidity, sample colour, and limited selectivity pose additional issues in such applications [32].

Electrochemical sensors have been implemented for different heavy metal monitoring. Normally, electrochemical detection methods are very sensitive. Nonetheless, long term monitoring using these methods is challenging because of limitations such as sensor drift, high maintenance cost, biofouling, and difficulty in analyzing complex matrices [33].

Analysis using optical detection systems minimizes fouling effects by avoiding the need for direct contact between sample and sensor. Colorimetric methods allow simple detection systems, therefore, making them suitable for portable heavy metal monitoring [34]. 

In the literature a wide range of different chromophoric dyes for heavy metal detection in water have been described [35,36,37,38,39]. Leucomalachite green (LMG) dye has been used for arsenic detection in environmental samples. In the procedure described by Revanasiddappa et al., arsenic is reacted with potassium iodate in order to liberate iodine. The liberated iodine in turn oxidises LMG to malachite green (MG) which in the presence of sodium acetate buffer forms a strong green colour. The intensity of the formed colour depends on the concentration of arsenic in the sample [40]. To date the LMG method has not been incorporated into a microfluidic detection system. 

Accordingly, this study aims to optimize a LMG method for cost effective and simple integration into a microfluidic detection system. LMG was selected due to the intense colour development in the visible region at 617 nm. Performance of the optimized method was evaluated in the laboratory on macro- and microscale. 

## 2. Results and Discussion 

### 2.1. Analytical Data

**Figure 1:**
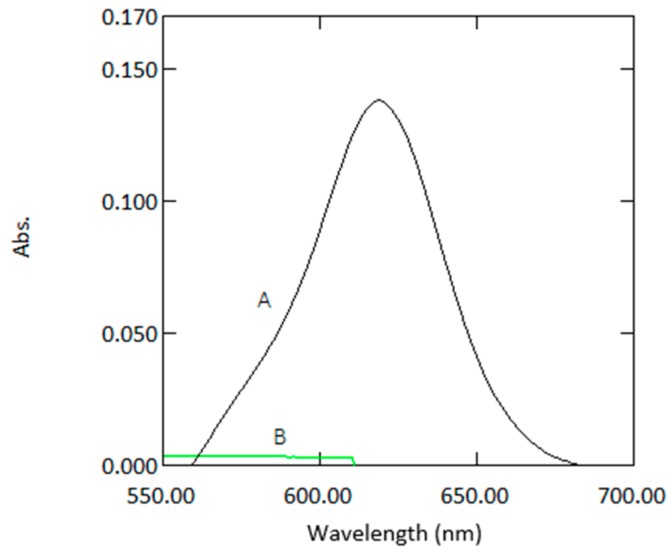
Absorption spectra of coloured species (1 µg mL^−1^ arsenic) versus reagent blank (A) and reagent blank versus double deionized water (B).

Absorption spectra of 1 µg mL^−1^ arsenic sample against reagent blank and reagent blank against double deionized water are shown in Figure 1. Beer’s law was obeyed in the range of 0.07–3 µg mL^−1^. The molar absorptivity coefficient was found to be 1.5 × 10^4^ L mol^−1^ cm^−1^. Sandell’s sensitivity was found to be 0.2 × 10^−2^ µg cm^−1^. The limit of detection (LOD = 3 se S^−1^) and the limit of quantification (LOQ = 10 se S^−1^, where se is the standard error of the calibration curve and S is the slope of the calibration curve) were found to be 0.19 and 0.64 µg mL^−1^, respectively. 

In comparison, Narayana et al. described a colorimetric arsenic detection method with a LOD of 0.022 µg mL^−1^ and linear range between 0.2 and 14 µg mL^−1^ [41]. Chakraboty et al. described an arsenic detection method with a LOD of 0.4 µg mL^−1^ and linear range between 0.4 and 12 µg mL^−1^ [42]. Alternatively, electrochemical methods have been utilized which have shown lower detection limits with broader linear ranges. For example, Lin et al. developed an arsenic detection assay based on G-quadropole complex with LOD of 4.5 ng mL^−1^ and linear range of 0.74–14.98 µg mL^−1^ [43]. Dai and Compton used electrodes coated with gold nanoparticles for determination of arsenic and achieved LOD of 5 ng mL^−1^ and a linear range of 1–180 ng mL^−1^ [44].

However, colorimetric methods should be considered for arsenic monitoring purposes as they have several advantages over electrochemical methods in terms of cost effectiveness, portability, and lower susceptibility to fouling. 

### 2.2. Path Length

**Figure 2:**
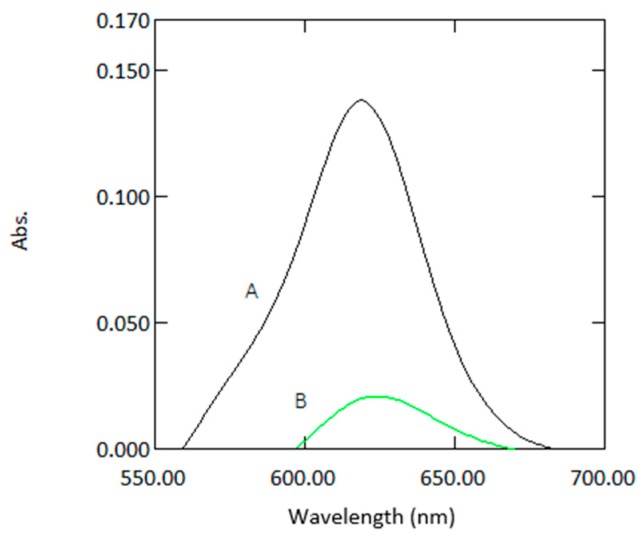
Absorption spectra of a sample containing 1µg mL^−1^ arsenic with reagents measured in 10 mm cuvettes (A) and 1 mm quartz cuvettes (B) against reagent blank.

**Figure 3:**
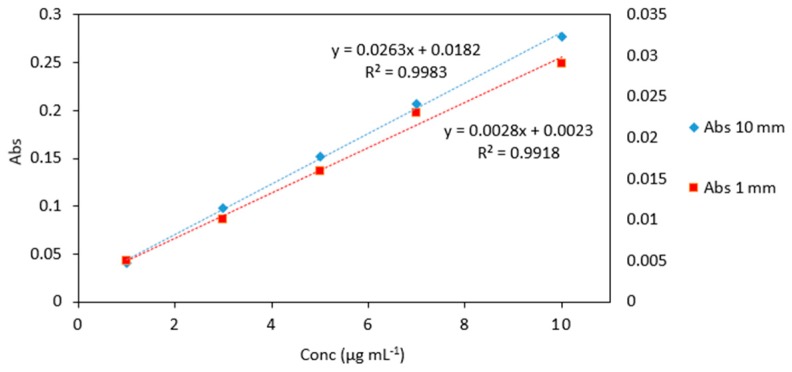
Comparison of arsenic standards (1–10 µg mL^−1^) measured in quartz cuvettes with 10 mm and 1 mm path lengths. Left vertical axes represents the absorbance of standards analyzed in 10 mm quartz cuvettes (blue markers). Right vertical axes represents the absorbance of standards analyzed in 1 mm quartz microcuvettes (red markers). All measurements were carried out in triplicate.

The absorbance values for 1 mm quartz cuvette measurements was 10 times lower than for standard cuvette measurements, as expected (Table 1 and Figure 2). As it can be seen from the calibration graphs (Figure 3) the analytical response was strong for samples measured in microcuvettes. The linearity compared to standard quartz cuvette measurements was good. The response signal and linearity obtained from the microcuvettes was strong which indicates that the leucomalachite green method is suitable for use in microfluidic detection systems. 

### 2.3. Time

**Figure 4:**
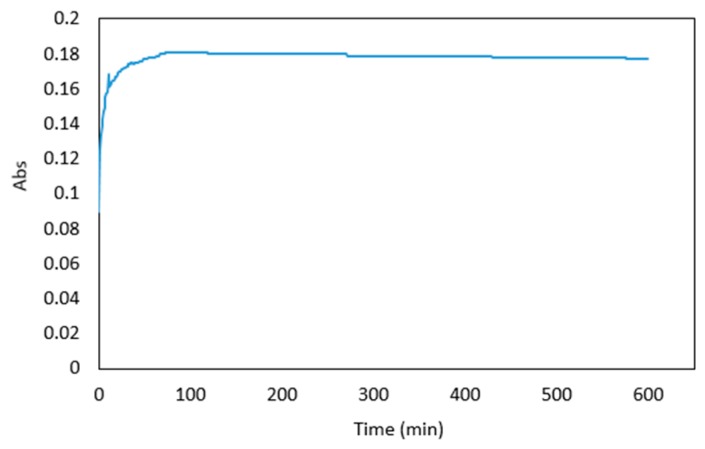
Absorbance of 1 μg mL^−1^ arsenic sample premixed with the reagents over 600 min.

The maximum absorbance was reached 73 min after the addition of the dye, however, the absorbance reached 95% of the maximum value within approximately 5 min. At this time the absorbance was sufficiently stable to allow a measurement to be taken, and a 5 min reaction time was used in subsequent experiments. After the maximum absorbance was observed, there was a gradual decrease in absorbance up to a time of 600 min (Figure 4). Overall, the colour stability was good and suitable for measurements in a microfluidic detection system.

### 2.4. Interference

Among the different species investigated. Fe (II) interfered with the leucomalachite green method (Table 2). Masking agents such as EDTA, citric acid, ascorbic acid and trimethylethanolamine were tested to overcome the interference, but it was found, that EDTA trimethylethanolamine, and citric acid themselves interfered with the leucomalachite green method. Iron interference was therefore masked by 1% ascorbic acid.

### 2.5. Optimization of Parameters

#### 2.5.1. Temperature

**Figure 5:**
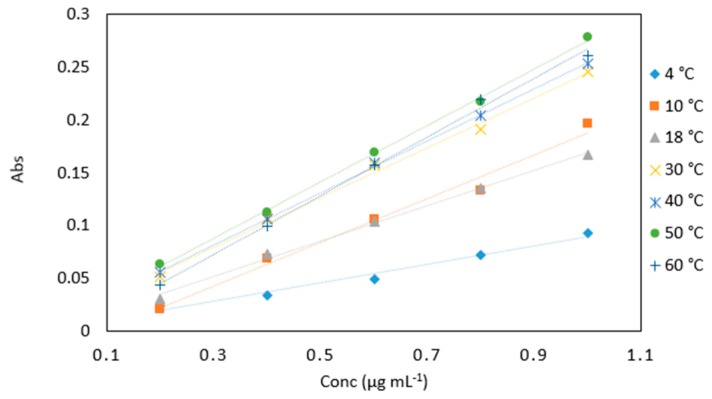
A comparison of arsenic samples (0.2–1 µg mL^−1^) analyzed at various incubation temperatures (4, 10, 18, 30, 40, 50 and 60 °C). All measurements were carried out in triplicate (*n* = 105).

The method performed best at 50 °C (Figure 5). For practical applications, carrying out the method at high temperatures would add to the cost and overall complexity of the method. The slope and linearity of 4 °C incubation temperature was low compared to the other temperatures (Table 3). It can, however, be concluded that the method has the potential to be applied in low temperature environments, and further examination of the kinetics of the reaction at low temperatures will be carried out. 

#### 2.5.2. pH

**Figure 6:**
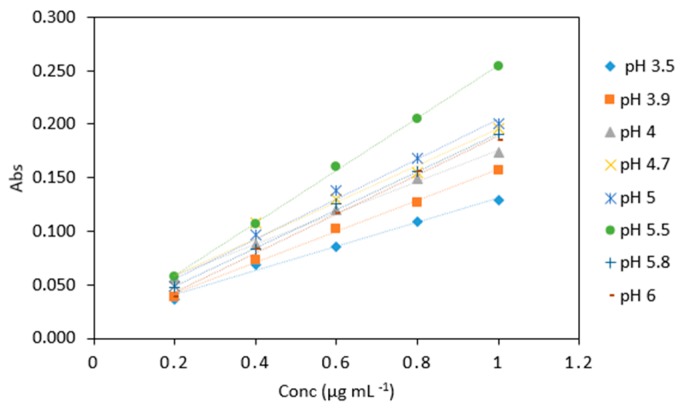
Comparison of arsenic samples (0.2–1 µg mL^−1^) analyzed using various sodium triacetate buffers (pH 3.5, 3.9, 4, 4.7, 5, 5.5, 5.8, 6). All measurements were carried out in triplicate (*n* = 120).

Table 4 shows the results obtained when arsenic samples were analyzed using different buffer pH values. The highest absorbance values were obtained when using pH 5.5 buffer (Table 4). Because of the analytical response buffer pH of 5.5 was found to be the optimum pH for the procedure (Figure 6) and used in subsequent experiments. Using ANOVA analysis, significant difference was found between the absorbances at different buffer pH (*p* < 0.05).

#### 2.5.3. Reagent Ratio

**Figure 7:**
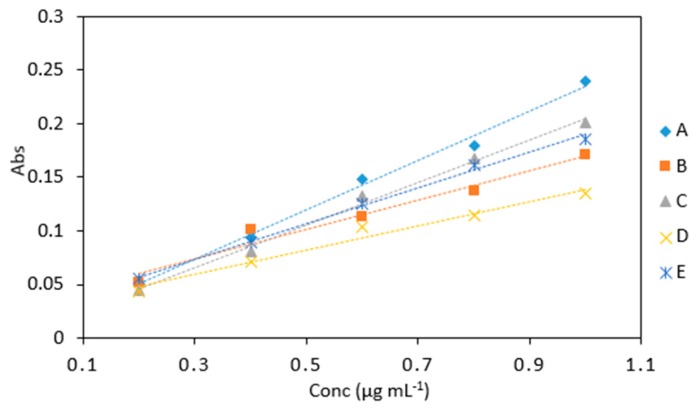
A comparison of arsenic samples (0.2–1 µg mL^−1^) analyzed using several reagent ratios: (A), 6 (As): 1(KIO_3_): 0.5 (1 M HCl): 0.5: (LMG): 2 (sodium triacetate buffer), (B), 6 (As): 2.5 (KIO_3_): 2.5 (0.2 M HCl): 2.5: (LMG and buffer), (C), 6 (As): 2.5 (KIO_3_ and 0.4 M HCl): 2.5 (LMG and buffer), (D), 2 (As): 2 (1% KIO_3_ and 0.4 M HCl): 2 (LMG and sodium triacetate buffer) and (E) 2 (As): 1(1% KIO_3_ and 0.4 M HCl): 1 (LMG and sodium triacetate buffer).

The statistical analysis showed that there was a significant difference between the absorbances obtained using different reagent ratios (*p* < 0.05). Reagent ratio A gave the best response as it had the highest slope (Figure 7) from all the reagent ratios tested and also the highest absorbance values (Table 5). However, for microfluidic detection system use reagent ratio D was chosen as it has the simplest reagent ratio. Small number of reagents is desirable for colorimetric method’s incorporation into microfluidic chip, as this reduces the fabrication costs. Therefore, this would simplify the design of the microfluidic detection system and overall device. 

#### 2.5.4. Reagent Stability

**Figure 8:**
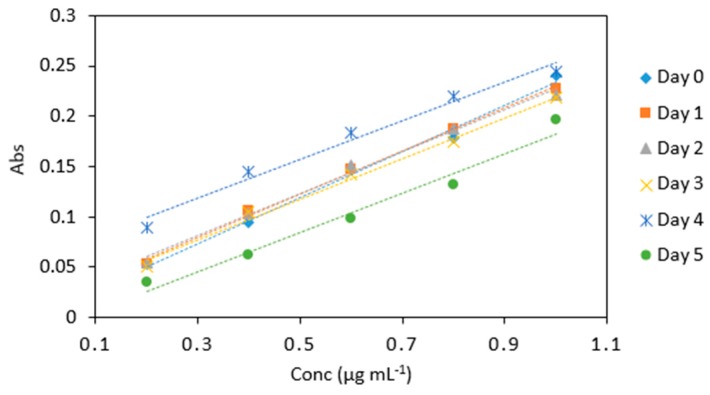
Stability of potassium iodate and hydrochloric acid mix in arsenic samples (0.2–1 µg mL^−1^) analyzed periodically over day 0, 1, 2, 3, 4, and 5. All measurements were carried out in triplicate (*n* = 90).

Decreasing absorbance values over time were noted for KIO_3_ and 0.4 M HCl mixture (Table 6 and Figure 8), however, the slope of the calibration line was relatively consistent in each case. The method yielded analytically useful calibration data over the time period studied and for implementation in a microfluidic device, the change in absolute absorbance values can be corrected for using a regular calibration protocol. 

**Figure 9:**
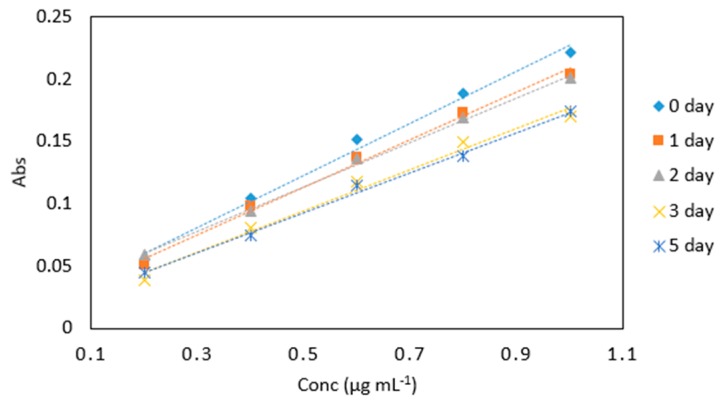
Comparison of arsenic samples (0.2–1 µg mL^−1^) analyzed periodically on day 0, 1, 2, 3 and 5 under the same conditions with the leucomalachite green dye and in sodium triacetate buffer (pH 5.5). All measurements were carried out in triplicate (*n* = 75).

A decrease in absorbance for sodium triacetate buffer and LMG dye mix was observed over a five day period (Table 7 and Figure 9), however, the slope of the calibration line was relatively consistent in each case. 

### 2.6. Environmental Samples

**Figure 10:**
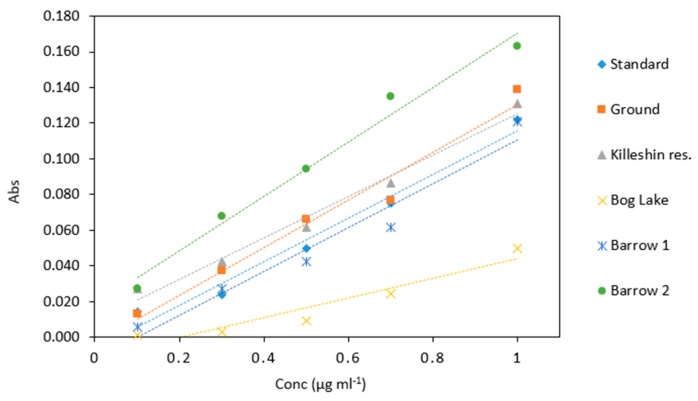
Comparison of arsenic samples (0.1–1 µg mL^−1^) analyzed in several water matrices. All measurements were carried out in triplicate (*n* = 90).

The highest absorbance values were observed in samples collected from the St Mullins site. The lowest absorbance was obtained from Bog Lake samples (Table 8). Water samples collected and analyzed from Killeshin reservoir and the River Barrow Carlow site had similar response to control samples (Figure 10). 

This would indicate that these water samples matrices did not contain high amounts of interfering substances. Statistical analysis revealed significant difference between Bog Lake and all the other water samples (*p* < 0.05). Also, significant difference was found between Barrow 2 and the other water sample matrices, except for control. The difference in absorbance values could be explained by factors such as sample colour and chemical composition. The Bog Lake sample was strongly coloured due to the presence of humic substances. From this it can be concluded that different water matrices have the potential to affect the result of the leucomalachite green method and this should be taken into account when designing the calibration protocol in any future analytical device. 

## 3. Materials and Methods 

### 3.1. Apparatus

A 1800 UV-visible spectrometer (Shimadzu, Canby, OR, USA) was used with Hellma (Mullheim, Germany) 10 mm and 1 mm quartz cuvettes for the absorbance measurements. A pH 20 pH meter (Hanna, Nusfalau, Romania) was used for pH measurements.

### 3.2. Reagents

All chemicals were of analytical grade and purchased from Sigma-Aldrich (Vale Road, Arklow, Co. Wicklow, Ireland), unless otherwise stated. Sodium meta-arsenite (NaAsO_2_), iron sulphate heptahydrate (FeSO_4_·7H_2_O) (Fisher Scientific, Leicestershire, UK), magnesium sulphate (MgSO_4_) (Fisher Scientific), potassium dihydrogen phosphate (KH_2_PO_4_), manganese sulphate monohydrate (MnSO4·H_2_O), and sodium nitrate (NaNO_3_)were used to prepare stock solutions at concentration 1000 µg mL^−1^ in double deionised water (HPLC grade). Working standards were prepared by serial dilutions. Acetic acid (99.8%) (Sharlab S.L., Barcelona, Spain) was used to adjust the pH. Trimethanolamine ((HOCH_2_CH_2_)_3_N), citric acid, (HOC(COOH)(CH2COOH)2) ascorbic acid (C6H8O6), potassium iodate (KIO_3_), leucomalachite green dye (C_6_H_5_CH[C_6_H_4_N(CH_3_)_2_]_2_), sodium triacetate trihydrate (C_2_H_3_NaO_2_·3H_2_O) were prepared by weighing out an appropriate amount and dissolving it in double deionised water. Hydrochloric acid (38%) (Sharlab S.L.) was used to prepare hydrochloric acid solutions with various concentrations in double deionised water. Double deionized water was used for dilution of reagents and samples.

### 3.3. Sample Preparation

Arsenic (As) sample (6 mL) was transferred to a glass vial. Potassium iodate (1%, 1 mL) and hydrochloric acid (1 M, 0.5 mL) were added, and the mixture was gently shaken and left for 2 min. Leuco malachite green dye was added (0.05%, 0.5 mL), followed by sodium triacetate buffer (13.6%, 2 mL). The mixture was gently shaken and left for 5 min. The absorbance was measured at 617 nm against reagent blank. 

### 3.4. Path Length

Effect of cuvette light path on absorbance was investigated. The procedure was carried out in standard 10 mm quartz cuvettes and micro cuvettes with 1 mm light path for 1–10 µg mL^−1^ arsenic concentration range. The experiment was carried out in triplicate. The average absorbance was calculated and showed in the result tables and calibration curves were plotted.

### 3.5. Time

The stability of the colour of the sample was tested over time. One µg mL^−1^ arsenic sample was analyzed for a time period of 600 min. The absorbance measurement was started after the addition of the dye. The procedure was carried out in triplicate.

### 3.6. Interference

The effect of various foreign species at µg mL^−1^ level on the determination of arsenic was examined. Various foreign ions with concentrations ranging from 100 to 200 µg mL^−1^ were introduced to 1 µg mL^−1^ arsenic sample. Tolerance limits of interfering agents were established at concentrations that do not cause more than 5% error in the absorbance values of arsenic at 1 µg mL^−1^. 

### 3.7. Optimization of Parameters

#### 3.7.1. Temperature

A range of different incubation temperatures (4–60 °C) were analyzed. Low temperatures were used in order to determine the viability of the method in low-temperature environments.

#### 3.7.2. pH

The effect of sodium triacetate buffer pH was studied using a range of different pH (3.7–7.3). Also, one-way analysis of variance (ANOVA, Single Factor) was applied to analyze the results.

#### 3.7.3. Reagent Ratio

The effect of combining different reagents and changing the reagent ratio was studied. The original ratio of the method was: 6 (As): 1(KIO_3_): 0.5 (1 M HCl): 0.5: (LMG): 2 (sodium triacetate buffer) (A). Firstly, the dye and the buffer were combined to give a reagent ratio: 6 (As): 2.5 (KIO_3_): 2.5 (0.2 M HCl): 2.5: (LMG and buffer) (B). Secondly, 1% KIO_3_ and HCl were combined to give reagent ratio of: 6 (As): 2.5 (KIO_3_ and 0.4 M HCl): 2.5 (LMG and buffer) (C). Thirdly, reagent ratio tested was: 2 (As): 2 (1% KIO_3_ and 0.4 M HCl): 2 (LMG and sodium triacetate buffer) (D). Fourthly, reagent the ratio assessed was: 2 (As): 1(1% KIO_3_ and 0.4 M HCl): 1 (LMG and sodium triacetate buffer) (E). Additionally, one-way analysis of variance (ANOVA, Single Factor) was used to analyze the results obtained from the different reagent ratios.

#### 3.7.4. Reagent Stability

The effect of reagent stability on the arsenic determination was investigated. 1% KIO_3_ and 0.4 M HCl reagent mixture was prepared and used for arsenic determination over 5 day time period. Over the course of the experiment fresh arsenic standards and dye and buffer reagent mixture was prepared daily.

The sodium triacetate buffer and LMG dye mixture was prepared and used for arsenic analysis over a 4 day time period. During this fresh arsenic standards and KIO_3_ and 0.4 M HCl mixture was prepared daily. 

### 3.8. Environmental Samples

Water samples were collected from Bog Lake, Co. Laois (pH 8.39), Killeshin water reservoir, Co. Laois (pH 7. 93), groundwater well Co. Laois (pH 7.4), St. Mullins, Co. Carlow (Barrow 1) (pH 7.31) and the River Barrow Carlow (Barrow 2) (pH 7. 27). All water samples were analyzed in triplicate. The sample matrices were analyzed using the leucomalachite green method in order to determine whether or not arsenic was present in concentrations detectable by the method. The different water matrices were then spiked with arsenic (0.03–20 µg mL^−1^) and appropriate dilutions were made. Prior to the analysis the water samples were filtered firstly using Whatman grade 1 filter paper and secondly with sterile 0.2 μm syringe filters. The pH of the water samples was adjusted to 5.5. The absorbances between different water matrices were compared. In addition, one-way analysis of variance (ANOVA, Single Factor) was used to analyze the results for the different water matrices. 

## 4. Conclusions

The leucomalachite green method proved to be a good candidate for deployment in microfluidic detection systems and arsenic detection in water. The method was optimized for integration into small scale detection systems. The optimum reaction conditions and other analytical parameters were evaluated. The method was found to be simple, reproducible, fast and robust. The reagent mixtures yielded the optimum results on the day of their preparation, with gradual decrease in absorbance noted over five days. Strong analytical response was obtained from 1 mm light path cuvettes indicating that the method would be suitable for use in small dimension microfluidic detection system. The optimized method was also cost effective as only a small number of reagents were required. The method yielded good results with simple 1 to 1 sample to reagent ratio which would be ideal for microfluidic detection applications. Following an investigation of method’s performance in different water samples, it was shown that the method is capable to determine arsenic in various water matrices. There is a potential for method’s application in waste water monitoring as well as arsenic detection in areas with particularly high arsenic levels. 

## Figures and Tables

**Table 1:** Average absorbance values for arsenic samples (0.2–1 µg mL^−1^) analyzed in two different types of cuvettes. Abs 1, SD 1 and % RSD 1 show the data for measurements carried out with 10 mm quartz cuvettes. Abs 2, SD 2 and % RSD 2 show the data for measurements in 1 mm quartz cuvettes. All measurements were carried out in triplicate (*n* = 30).

Conc (µg mL^−1^)	Abs 1	Abs 2	SD 1	SD 2	% RSD 1	% RSD 2
1	0.041	0.005	0.003	0.001	6.089	16.33
3	0.098	0.01	0.002	0	2.365	0
5	0.152	0.016	0.001	0	0.379	2.886
7	0.207	0.023	0.002	0.003	0.746	12.298
10	0.277	0.029	0.003	0	1.083	1.607

**Table 2:** Effect of foreign species on the determination of arsenic (III) (1 µg mL^−1^).

Interferents	Tolerance Limit (μg mL^−1^)
Mn, Mg, PO_4_, NO_3_	100
Fe (II)	0.1
EDTA	3800
Citric acid	100,000
Trimethylethanolamine	450

**Table 3:** Average absorbance values of arsenic samples (0.2–1 µg mL^−1^) analyzed at various incubation temperatures (4, 10, 18, 30, 40, 50 and 60 °C). All measurements were carried out in triplicate (*n* = 105).

	Temperature						
Conc (µg mL^−1^)	4 °C	10 °C	18 °C	30 °C	40 °C	50 °C	60 °C
0.2	0.024	0.021	0.030	0.051	0.056	0.063	0.044
0.4	0.034	0.069	0.073	0.107	0.106	0.113	0.099
0.6	0.049	0.106	0.104	0.157	0.159	0.169	0.157
0.8	0.072	0.133	0.135	0.191	0.204	0.217	0.220
1	0.093	0.197	0.167	0.246	0.254	0.279	0.261
Slope	0.088	0.208	0.168	0.243	0.253	0.273	0.270
R^2^	0.977	0.983	0.995	0.996	0.999	0.999	0.997

**Table 4:** Average absorbance of arsenic samples (0.2–1 µg mL^−1^) analyzed with various sodium triacetate buffer pH. All measurements were carried out in triplicate (*n* = 120).

Conc (µg mL^−1^)	pH 3.5	pH 3.9	pH 4	pH 4.7	pH 5	pH 5.5	pH 5.8	pH 6
0.2	0.037	0.039	0.057	0.048	0.057	0.057	0.048	0.038
0.4	0.068	0.074	0.088	0.108	0.106	0.106	0.083	0.084
0.6	0.085	0.102	0.120	0.13	0.161	0.161	0.125	0.116
0.8	0.109	0.127	0.149	0.154	0.205	0.205	0.155	0.156
1	0.129	0.157	0.174	0.196	0.254	0.254	0.190	0.185
Slope	0.126	0.081	0.168	0.171	0.200	0.253	0.188	0.188
R^2^	0.983	0.989	0.979	0.968	0.990	0.999	0.995	0.997

**Table 5:** Average absorbance of arsenic samples (0.2–1 µg mL^−1^) analyzed using various reagent ratios: (A), 6 (As): 1(KIO_3_): 0.5 (1 M HCl): 0.5: (LMG): 2 (sodium triacetate buffer), (B), 6 (As): 2.5 (KIO_3_): 2.5 (0.2 M HCl): 2.5: (LMG and buffer), (C), 6 (As): 2.5 (KIO_3_ and 0.4 M HCl): 2.5 (LMG and buffer), (D), 2 (As): 2 (1% KIO_3_ and 0.4 M HCl): 2 (LMG and sodium triacetate buffer) and (E) 2 (As): 1(1% KIO_3_ and 0.4 M HCl): 1 (LMG and sodium triacetate buffer). All measurements were carried out in triplicate (*n* = 75).

	Reagent Ratio				
Conc (µg mL^−1^)	A	B	C	D	E
0.2	0.053	0.052	0.045	0.043	0.056
0.4	0.094	0.101	0.081	0.071	0.089
0.6	0.148	0.113	0.132	0.104	0.126
0.8	0.179	0.137	0.168	0.114	0.162
1	0.240	0.171	0.201	0.135	0.186
Slope	0.232	0.160	0.203	0.114	0.167
R^2^	0.996	0.957	0.996	0.971	0.996

**Table 6:** Average absorbance values for arsenic samples (0.2–1 µg mL^−1^) analyzed periodically on day 0, 1, 2, 3, 4 and 5 under the same conditions with the same potassium iodate and hydrochloric acid mix. All measurements were carried out in triplicate (*n* = 90).

	Time (Days)					
Conc (µg mL^−1^)	0	1	2	3	4	5
0.2	0.089	0.053	0.055	0.053	0.051	0.036
0.4	0.145	0.106	0.104	0.094	0.105	0.062
0.6	0.184	0.147	0.151	0.148	0.142	0.098
0.8	0.220	0.187	0.188	0.179	0.175	0.132
1	0.244	0.228	0.221	0.240	0.218	0.196
Slope	0.193	0.216	0.208	0.233	0.202	0.186
R^2^	0.978	0.997	0.993	0.995	0.993	0.978

**Table 7:** Average absorbance for arsenic samples (0.2–1 µg mL^−1^) analyzed periodically on day 0, 1, 2, 3, 4 and 5 under the same conditions with the same dye and buffer mix. All measurements were carried out in triplicate (*n* = 75).

	Time (Days)				
Conc (µg mL^−1^)	0	1	2	3	5
0.2	0.055	0.051	0.059	0.039	0.044
0.4	0.104	0.098	0.094	0.081	0.075
0.6	0.151	0.137	0.136	0.118	0.115
0.8	0.188	0.173	0.168	0.149	0.138
1	0.221	0.204	0.201	0.170	0.174
Slope	0.223	0.191	0.178	0.166	0.161
R^2^	0.996	0.994	0.977	0.986	0.996

**Table 8:** The average absorbance of arsenic samples (0.1–1 µg mL^−1^) analyzed in different water matrices. The measurements were carried out in triplicate (*n* = 90).

	Water Samples					
Conc (µg mL^−1^)	Control	Ground	Killeshin Res.	Bog Lake	Barrow 1	Barrow 2
0.1	0.014	0.013	0.027	0.001	0.006	0.027
0.3	0.024	0.037	0.042	0.003	0.027	0.068
0.5	0.050	0.066	0.061	0.009	0.042	0.094
0.7	0.075	0.077	0.087	0.024	0.061	0.135
1	0.122	0.139	0.131	0.050	0.121	0.163
Slope	0.114	0.135	0.104	0.072	0.123	0.090
R^2^	0.996	0.978	0.996	0.996	0.955	0.999
